# Public service motivation and career choice intentions of social work students: the roles of altruistic motivation and professional values

**DOI:** 10.3389/fpsyg.2025.1517457

**Published:** 2025-02-27

**Authors:** Zheng-Xin Hu, Kai-Peng Gan, Guo-Yuan Sun, Qiu Wang

**Affiliations:** ^1^School of Ethnology and Sociology, Yunnan University, Kunming, China; ^2^School of Low and Political Science, Yunnan University of Finance and Economics, Kunming, China; ^3^College of Innovation and Management, Suan Sunandha Rajabhat University, Bangkok, Thailand

**Keywords:** public service motivation, career choice intentions, altruistic motivation, professional values, social work

## Abstract

The shortage and high turnover of social work professionals in China pose a major challenge to social governance and societal well-being, making it crucial to understand the factors influencing social work students’ career choices. Based on a purposive sampling method, between April and June 2023, a sample of 624 social work students was collected from universities in three provinces of China (Guangdong, Jiangxi, and Yunnan). The present study employs the SPSS PROCESS macro to examine the relationship between public service motivation (PSM) and career choice intentions, exploring how altruistic motivation and professional values influence the impact of PSM on career decisions. The results indicate that PSM and its dimensions strongly affect social work students’ career intentions, with altruistic motivation partially mediating this effect. Professional values positively moderate PSM’s impact, strengthening its influence on career choices. These findings provide valuable insights for social work education, guiding career strategies to reduce turnover and support the profession’s sustainable development.

## 1 Introduction

Social work has long been recognized as a foundational discipline for cultivating professionals in the field of social services, with the goal of developing students who possess solid theoretical knowledge and practical skills to serve those in need (Paat, 2016). As a result, social work students often exhibit strong altruistic values, prioritizing careers that allow them to help others. However, with the continued development of the market economy, there has been a shift in the career values of some social work students, with a growing focus on financial rewards, social status, and job stability. This shift has led to complex changes in their career choices, particularly regarding employment in the public and non-profit sectors. Currently, over 300 colleges in China offer social work programs, and the number of social work graduates continues to grow each year. Yet, there is increasing concern about the low retention of social work students in the public service sector, as many graduates either avoid or quickly leave these roles after entering the workforce. Some studies indicate that social work students are increasingly prioritizing personal interests over traditional altruistic values in career decisions (Chu, 2020). According to data from the Ministry of Education (2022), fewer than 30% of China’s annual 30,000 social work graduates choose careers in the nonprofit sector. This shortage of social work professionals hinders the field’s growth and undermines service quality in public and nonprofit sectors. Understanding the factors influencing career choices among social work students is vital for the profession’s sustainable development.

Previous studies on college students’ career choice intentions have primarily focused on factors such as career opportunities, internship environment, and student loan debt (Nguyen et al., 2023; Anyango et al., 2024; Paulsen, 2024), with limited attention to motivational factors. Public service motivation (PSM) has emerged as a growing area of research, attracting significant academic interest. While several empirical studies have explored the link between PSM and career intentions, most have focused on employed individuals, where work-related norms and values may reduce PSM’s impact (Sanabria-Pulido, 2018). Although PSM theory suggests that PSM influences sectoral employment choices, empirical findings remain inconsistent (Perry and Wise, 1990). Some studies find no significant relationship between PSM and career intentions (Lee and Choi, 2016), while others suggest PSM predicts students’ intention to enter the public sector (Holt, 2018; Asseburg and Homberg, 2020; Hinna et al., 2021). These mixed results highlight the need for further investigation into the relationship between PSM and career choice intentions.

Furthermore, most existing studies have examined the direct impact of PSM in Western contexts, with limited research on the mechanisms underlying this relationship, especially in non-Western settings. Altruistic motivation, as an intrinsic driving force, aligns with the normative elements of PSM and may affect career choices by motivating individuals to prioritize careers that benefit others or society. Prior research mainly focused on the mediating role of interpersonal citizenship behaviours, charitable giving, volunteering, and so on (Houston, 2006; Pandey et al., 2008). However, some scholars argue that PSM is a precursor to altruistic motivation, as the essence of PSM lies in helping others and serving the public interest (Gans-Morse et al., 2019; Zubair et al., 2021). Individuals with stronger PSM are more likely to possess higher levels of altruistic motivation, making them more inclined to pursue careers in the public sector or nonprofit organizations in order to better serve others and society. Additionally, professional values, which represent an individual’s core beliefs and attitudes toward work, are central to career choice decisions. Schein’s (1985) Career Anchor Theory suggests that career anchors—values individuals prioritize when making difficult career choices—serve as focal points for professional development. Some scholars have explored the mediating roles of variables such as relational work and collectivist professional values in the association between PSM and career choice (Choi, 2017; Miller-Mor-Attias and Vigoda-Gadot, 2022). However, little attention has been given to the potential impact of professional values. In fact, as one of the potential outcome variables of PSM, professional values have a significant direct impact on individuals’ career choices, particularly in determining whether they pursue the economically driven business sector or opt for the nonprofit sector (Choi, 2017; Ni et al., 2022), which offers greater opportunities to serve society. Therefore, both altruistic motivation and professional values may also play a critical role in shaping the relationship between PSM and career choice intentions.

**Figure 1:**
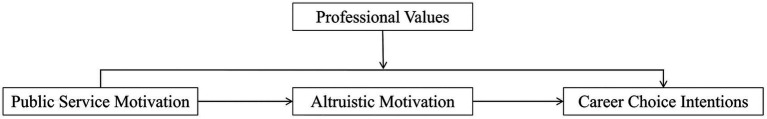
Research model.

The social work profession originated in Western countries and is deeply rooted in Western culture. It wasn’t until 1912 that the profession began to spread and develop in China, albeit in a somewhat indirect manner. In recent years, however, due to market economy factors and employment policy trends, some Chinese universities have begun to discontinue their social work programs. How to localize the development of social work has become a pressing issue for Chinese universities and scholars to address. At the same time, both altruistic motivation and public service motivation are concepts rooted in Western culture, with their origins traceable to Perry (1996). Whether these concepts have equivalents in traditional Chinese culture is a question worth pondering. As a result, it becomes even more crucial to explore whether public service motivation and altruistic motivation significantly affect the career choices of social work students within the context of Chinese culture. Based on these theoretical considerations and previous research, the present study incorporates public service motivation, altruistic motivation, and professional values into a career choice framework, exploring the direct impact of public service motivation on social work students’ career choices, as well as the mediating roles of altruistic motivation and professional values in this relationship (Figure 1). This research contributes to the academic discussion of career choice motivations among social work students and provides theoretical insights for strengthening the development of social work professionals in China.

## 2 Literature review and hypotheses

### 2.1 PSM and career choice intentions

The concept of Public Service Motivation (PSM) was first introduced by American scholars Perry and Wise (1990), who defined it as “an individual’s inclination to respond to motives grounded primarily or uniquely in public institutions or collective organizations.” They identified three foundational motives that comprise PSM: rational, normative, and affective motives. Perry (1996) expanded on this framework by categorizing PSM into four measurable dimensions: attraction to public policy-making, commitment to the public interest, compassion, and self-sacrifice, which serve as indicators of an individual’s level of PSM. Over time, the concept of PSM has been further developed. For instance, Vandenabeele (2007) defined PSM as “beliefs, values, and attitudes that transcend beyond personal and organizational interests, focusing on the broader political body and motivating individuals to take appropriate actions when necessary.” Today, PSM is widely recognized as a key factor in explaining employee work behavior, with empirical support across both public and private sectors (Gan et al., 2020; Wang et al., 2024; Nguyen et al., 2024).

The self-determination theory is particularly influential in research linking PSM to career choice intentions. According to this theory, individuals make behavioral choices based on their personal needs and surrounding context. It emphasizes the role of intrinsic motivation in guiding individuals toward self-determined activities, with those driven by intrinsic factors being more likely to engage in behaviors that align with their values (Ryan and Deci, 2024). In the context of PSM and career choice intentions, individuals with high levels of PSM are intrinsically motivated to pursue public service roles, as their personal values resonate with the mission of public organizations. This intrinsic motivation enhances their sense of autonomy and competence, which, in turn, increases their likelihood of choosing a career in the public sector (Holt, 2018). A body of empirical research has consistently demonstrated a positive correlation between PSM and the intention to work in the public or nonprofit sectors. For example, Hinna et al. (2021) found that PSM significantly influenced Italian undergraduate economics students’ willingness to enter the public sector. Similarly, Korac et al. (2019) identified PSM as a significant factor in driving public sector employment preferences at the individual level. Bright (2016) confirmed that individuals with higher PSM are more inclined to pursue careers in the public sector, and that PSM has a stronger predictive power for career choices in the nonprofit sector than in the government sector. Asseburg and Homberg (2020) found that public service motivation is the main driver of public sector attractiveness. Piatak (2016) also showed that graduate students with higher levels of PSM are more likely to engage in volunteerism and pursue work in government or nonprofit organizations. Additionally, research examining the various dimensions of PSM has highlighted that some of these dimensions, such as commitment to the public interest or self-sacrifice, significantly influence college students’ intentions to choose public sector careers, further supporting the causal relationship between PSM and career choice (Ballart and Rico, 2018). Based on this literature, the following hypothesis is proposed:

**Hypothesis 1**: PSM is positively correlated with career choice intentions.

### 2.2 The mediating role of altruistic motivation

Auguste Comte was the first to introduce the term “altruistic behavior” into moral theory, arguing that humans are driven by both selfish and altruistic impulses and can act for reasons beyond their self-interest. In psychological terms, altruistic behavior is generally understood as voluntarily helping others without expecting a reward, with altruism representing the idea of helping others at a personal cost. Altruistic motivation and altruism are often described as motivational dimensions (Sibbald and Beagan, 2024). When applied to social work, the concept of altruism acquires professional significance, serving as a core value that social workers should uphold to establish their professional authority. The need for self-sacrifice for the betterment of others is a fundamental aspect of the profession’s mission.

Much of the academic research exploring the link between altruistic motivation and career choice intentions is rooted in the Theory of Planned Behavior. This theory posits that an individual’s behavioral intention, driven by motivation, influences their actions. When behavioral intention reaches a certain threshold, it prompts corresponding behavior (Ajzen, 1985). In a pioneering study, Hungarian scholars Győrffy et al. (2016) conducted a survey among 733 students, discovering that altruistic motivation was the primary factor driving career choices. Li (2018) also emphasized that altruism is a key driver for medical students’ career choices. More recent empirical studies support the idea that altruistic motivation significantly predicts career choice intentions (Pfarrwaller et al., 2022; Ikram et al., 2022). Although the majority of studies have focused on medical students, altruism also plays a central role in social work, with evidence suggesting that altruistic motivation is an important predictor of social work students’ choices to pursue careers in the nonprofit sector (Warde, 2009). In exploring the relationship between PSM and altruistic motivation, some scholars equate the two, viewing PSM as a “special form of altruistic motivation” (Bright, 2008). However, others argue that altruism generally relates to individuals’ motivations to help others in private spheres, while PSM specifically addresses the call to public service (Piatak and Holt, 2020). In this view, altruism is a one-dimensional concept, while PSM is multidimensional. Despite these discussions, few scholars have directly explored the connection between the two. According to Social Norm Theory, altruistic behavior is shaped by social culture and norms, with individuals internalizing societal values to form altruistic motivations (Cialdini and Goldstein, 2004). The normative foundation of PSM aligns with the social responsibility and moral obligation that underpin altruistic motivation. As a result, individuals with high PSM tend to have a stronger identification with and willingness to comply with social norms, which enhances their altruistic behavior (Meyer-Sahling et al., 2019).

Empirical studies have supported the idea that PSM positively affects altruistic motivation. For instance, Vandenabeele (2007) found that PSM increases the frequency and intensity of altruistic behavior by strengthening individuals’ sense of social responsibility. Recent research also highlights the significant positive impact of PSM on altruistic motivation. Gans-Morse et al. (2019) found that PSM is a key predictor of altruistic motivation, and Zubair et al. (2021) confirmed its positive effect on civil servants’ altruistic behaviors. A cross-cultural study by Kim et al. (2013) demonstrated that public sector employees with high PSM exhibit higher levels of altruistic behavior, regardless of cultural context. This suggests that the positive influence of PSM on altruistic motivation is widely applicable. Additionally, some scholars argue that PSM enhances intrinsic motivation by fulfilling individuals’ self-actualization needs, leading them to feel a greater sense of achievement and self-worth through public service. This reinforcement of intrinsic motivation further strengthens their altruistic behavior in both work and personal life (Wright et al., 2016).

Prior research has shown that altruistic motivation may mediate the relationship between PSM and career choice intentions. For example, Pandey et al. (2008) found that PSM influences career choices through altruistic behaviors such as interpersonal citizenship behaviors. Similarly, Houston (2006) verified that altruistic behaviours like charitable giving and volunteering, mediate the relationship between PSM and public service career choices. Based on this body of literature, it can be concluded that PSM enhances altruistic motivation, which in turn influences career choice intentions. Therefore, the following hypothesis is proposed:

**Hypothesis 2**: Altruistic motivation mediates the relationship between PSM and career choice intentions.

### 2.3 The moderating role of professional values

Super (1973) defined professional values as “goals and attributes related to work, representing an individual’s intrinsic needs and the characteristics or attributes of work that one seeks while engaging in activities.” Building on empirical research, he classified professional values into three dimensions: intrinsic values, extrinsic values, and extrinsic rewards.

The relationship between professional values and career choice intentions is rooted in the idea that careers are driven by personal values. Individuals are more likely to choose occupations that align with their professional values (Choi, 2017). According to Super’s (1973) Career Choice Theory, people tend to seek occupations and work environments that are consistent with their values and attitudes, thus fulfilling their intrinsic needs and value beliefs. Scholars have argued that professional values are dynamic, reflecting individuals’ hopes and expectations for their future work, and directly influencing career choices (Alkaya et al., 2018). A significant body of research has shown that professional values provide criteria for job selection and are a crucial factor in predicting individuals’ career intentions. For instance, Ni et al. (2022) found that career stability and prestige values in professional values significantly affected college students’ intentions to choose civil service positions. Choi (2017) demonstrated that students who prioritize altruistic professional values are more likely to select careers in the public or nonprofit sectors. Abrahamsen (2015) observed that nursing undergraduates’ choice of clinical fields after graduation was related to their professional values, with those placing more importance on altruistic values being more inclined to pursue general hospital nursing.

Prior research has suggested that the relationship between PSM and career choice intentions may be moderated by other factors. Wright and Christensen (2010) proposed that time could be a potential moderating variable in the connection between PSM and employees’ sector choices. Choi (2017) demonstrated that relational work could moderate the relationship between PSM and an individual’s decision to choose the public sector. Additionally, research has found that individuals who prioritize intrinsic values (such as collectivist professional values) early in their careers are more likely to be drawn to public service roles that align with these values. Students with higher intrinsic professional values, whose values closely align with the public sector, tend to exhibit stronger PSM over time (Miller-Mor-Attias and Vigoda-Gadot, 2022). For individuals prioritizing professional values related to social contribution and public interest, the positive impact of PSM on career choice intentions is likely stronger. This is because their professional values are highly consistent with the intrinsic motivations of PSM, making them more likely to pursue public service careers (Wright and Christensen, 2010). Based on the above discussions, this paper proposes the following hypothesis:

**Hypothesis 3**: Professional values positively moderate the relationship between PSM and career choice intentions.

## 3 Methods

### 3.1 Participants and sample

To enhance the representativeness of the sample, the present study adopted a purposive sampling method for the selection of survey areas and sampling subjects. First, one province was selected from each of China’s eastern, central, and western regions (Guangdong, Jiangxi, and Yunnan). Then, a comprehensive university offering both undergraduate and graduate social work programs was chosen in each province. In the selected survey universities, simple random sampling was used for data collection, with each university distributing 250–300 questionnaires. A total of 713 questionnaires were distributed between April and June 2023, with 688 completed questionnaires returned. Data for this study were collected from full-time undergraduate and graduate social work students who were from 18 provinces and regions across China. Participants were informed about the voluntary and anonymous nature of the survey prior to completing the questionnaire. The questionnaire included a major selection item as one of the criteria for excluding invalid responses. After excluding incomplete and invalid responses, as well as non-social work majors, 624 valid samples remained for analysis. Of the valid sample, 153 (24.5%) were male and 471 (75.5%) were female. The majority of respondents (95.2%) were under 25 years old. More than half of the participants came from rural households, comprising 67.1% of the sample. Additionally, only 33.7% of the respondents reported that their families’ socioeconomic status was classified as middle class or higher.

### 3.2 Measures

Authoritative scales were used to assess the variables used in this study. Respondents answered the items on a five-point Likert scale, ranging from 1 (Very Disagree) to 5 (Very Agree).

#### 3.2.1 Public service motivation

We referred to the Chinese translated version of a PSM scale developed by Perry (1996) and semantically adjusted it to the real context. The scale consists of 17 items with four dimensions. One of the items is, “Serving the public makes me feel good, even without compensation.” The Cronbach’s alpha for this scale is 0.816.

#### 3.2.2 Altruistic motivation

We evaluated students’ altruistic motivation levels utilizing a four-item scale developed by Siem and Stürmer (2018). It includes statements such as “I like doing things that can help others.” The Cronbach’s alpha for this scale is 0.834.

#### 3.2.3 Professional values

We measured the dimension of altruistic professional values using Choi’s (2017) scale, which includes two items: “Helping others in my community or those around me” and “Working to correct social and economic inequalities.” Higher scores suggest a greater emphasis on altruistic professional values. The Cronbach’s alpha for this scale is 0.787.

#### 3.2.4 Career choice intentions

We employed a four-item Career Choice Scale developed by Ashari et al. (2019) for measurement. To align with the employment characteristics of social work students, we adapted some of the items. For example, “The skills area will be my priority when choosing a career” was modified to “The public sector or nonprofit organizations will be my priority when choosing a career,” and “I will choose a career in the skills area” was modified to “I will choose a career in the public sector or nonprofit organizations.” Higher scores indicate a greater inclination to choose employment in the public or nonprofit sectors. The Cronbach’s alpha for this scale is 0.801.

### 3.3 Analytical strategy

This study first conducted descriptive statistical analysis, correlation analysis, common method bias testing, and robustness testing using SPSS version 27. Then, to test the fit of the hypothesized model, confirmatory factor analysis was performed using AMOS 24. Finally, we employed the SPSS PROCESS macro to test the mediating effect of altruistic motivation (Model 4) and the moderating effect of professional values (Model 1), respectively. Furthermore, we plotted the interaction plots to further explain the results of the hypothesis testing.

## 4 Results

### 4.1 Testing of common method bias

Since the data in this study were gathered using self-reported questionnaires, there is a possible risk of common method bias. To address this, we performed Harman’s single-factor test on the relevant items. According to the Harman single-factor test method, if one factor explains a majority of the variance (over 50%) in the factor analysis results, common method bias may be present. The findings revealed that the variance explained by the first factor of the hypothesized model was 34.434% (less than 50%), indicating that common method bias is not an issue in this research.

### 4.2 Confirmatory factor analysis

To test the fit of the hypothesized model, AMOS 24 was used for evaluation. This study primarily uses fit indices such as *χ*^2^/df (less than 5), CFI (greater than 0.90), TLI (greater than 0.90), and RMSEA (less than 0.08) as the judgment criteria for Confirmatory Factor Analysis (CFA). Table 1 illustrates that the four-factor model of the hypothesized model demonstrates good fit compared to several other models (*χ*^2^/df = 3.127; CFI = 0.928, TLI = 0.910, RMSEA = 0.058). As an example, the one-factor model shows a poor fit (*χ*^2^ (324) = 3613.769, *p* < 0.001; CFI = 0.602, TLI = 0.569, RMSEA = 0.128).

**Table 1:** Results for confirmatory factor analysis.

Model	*χ* ^2^	df	*χ*^2^/df	CFI	TLI	RMSEA	Δ*χ*^2^ (Δdf)
Hypothesized four-factor model	875.578	280	3.127	0.928	0.910	0.058	
Three-factor model							
Combining PSM with AM	2951.527	321	9.195	0.682	0.652	0.115	2075.949 (41)
Combining PSM with PV	2743.774	321	8.548	0.707	0.680	0.110	1868.196 (41)
Combining PSM with CCI	3031.933	321	9.445	0.672	0.642	0.116	2156.355 (41)
Two-factor model(combining PSM, AM, and PV)	3166.285	323	9.803	0.656	0.627	0.119	2290.707 (43)
One-factor model (combining all constructs)	3613.769	324	11.154	0.602	0.569	0.128	2738.191 (44)

*N = 624. PSM, public service motivation; AM, altruistic motivation; PV, professional values; CCI, career choice intentions. TLI, Tucker-Lewis index; CFI, comparative fit index; RMSEA, root mean square error of approximation.*

### 4.3 Correlation analysis

Table 2 presents the correlation coefficients among the variables used in this study. The results indicate that PSM is significantly and positively associated with altruistic motivation (r = 0.541, *p* < 0.01), professional values (r = 0.486, *p* < 0.01), and career choice intentions (r = 0.430, *p* < 0.01), respectively. Furthermore, altruistic motivation has a significant and positive correlation with career choice intentions (r = 0.476, *p* < 0.01). Similarly, there is a significant positive association between professional values and career choice intentions (r = 0.352, *p* < 0.01).

**Table 2:** Means, standard deviations, and correlations among the study variables.

Variable	1	2	3	4	5	6	7	8
1.Gender	1							
2.Age	0.066	1						
3.Household registration	0.030	−0.016	1					
4.Families’ socioeconomic status	−0.028	0.025	0.391**	1				
5.PSM	0.016	0.029	−0.050	0.012	(0.816)			
6.Altruistic motivation	0.005	0.001	0.029	0.045	0.541**	(0.834)		
7.Professional values	0.018	−0.059	−0.019	−0.016	0.486**	0.374**	(0.787)	
8.Career choice intentions	−0.015	−0.027	−0.054	−0.049	0.430**	0.476**	0.352**	(0.801)
Mean	0.245	1.710	0.329	2.200	3.498	3.928	3.767	3.676
SD	0.431	0.548	0.470	0.785	0.405	0.528	0.769	0.597

*N* = 624, *p < 0.05, **p < 0.01. SD, standard deviation. Figures in the bracket represent Cronbach’s Alpha Values.

### 4.4 Hypothesis testing

This study used multiple linear regression models to test the impact of PSM and its dimensions on social work students’ career choice intentions. As shown in Table 3, PSM has a significant positive effect on career choice intentions (b = 0.635, *p* < 0.001); that is, the stronger the PSM of social work students, the more they tend to choose employment in the public or nonprofit sector, thus supporting Hypothesis 1. In addition, each of the four dimensions of PSM (attraction to public policy-making, commitment to the public interest, compassion, and self-sacrifice) has a significant positive effect on career choice intention (b = 0.174, *p* < 0.001; b = 0.428, *p* < 0.001; b = 0.445, *p* < 0.001; b = 0.320, *p* < 0.001).

**Table 3:** Regression analysis and robustness testing.

Variables	Career choice intentions					
	OLS Regression					Binary Logistic Regression
	Model 1	Model 2	Model 3	Model 4	Model 5	Model 6
Constant	1.617***	4.202***	2.195***	2.027***	2.776***	−0.187
Gender	−0.028	−0.023	−0.012	−0.003	−0.065	−0.063
Age	−0.040	−0.032	−0.061	−0.032	−0.026	−0.034
Household registration	−0.017	−0.033	−0.035	−0.027	0.011	−0.030
Families’ socioeconomic status	−0.037	−0.021	−0.022	−0.027	−0.047	−0.009
PSM	0.635***					0.313***
Aattraction to public policy-making		0.174***				
Ccommitment to the public interest			0.428***			
Compassion				0.445***		
Self-sacrifice					0.320***	
*R* ^2^	0.189	0.051	0.177	0.166	0.156	0.111

*N* = 624, *p < 0.05, **p < 0.01, and ***p < 0.001.

In order to test the robustness of the above results, this study employed a binary logistic regression model to convert the dependent variable of career choice intentions into a binary variable (the options “very disagree,” “comparatively disagree,” “general” were assigned 0 points, and “agree” and “very agree” were assigned 1 point) for robustness testing. The findings show that PSM still has a significant positive effect on social work students’ career choice intention (b = 0.313, *p* < 0.001), which is consistent with the results of Model 1, hence demonstrating strong robustness.

To examine the mediating role of altruistic motivation, we utilized the SPSS PROCESS macro and selected Model 4 to evaluate and calculate the 95% confidence interval of the mediation effect (Bootstrap sample size = 5,000). As shown in Table 4, Models 1 and 3 demonstrate that PSM exerts a significant positive effect on altruistic motivation (b = 0.709, *p* < 0.001) and that altruistic motivation significantly positively impacts career choice intentions (b = 0.395, p < 0.001). When altruistic motivation is included as a mediating variable in Model 3, PSM still has a significant positive effect on career choice intentions, but the coefficient of effect is reduced (b = 0.355, p < 0.001). These findings suggest that altruistic motivation partially mediates the relationship between PSM and social work students’ career choice intentions (indirect effect = 0.280, 95% CI: [0.188, 0.374]). Consequently, Hypothesis 2 is confirmed.

**Table 4:** Results of mediating hypotheses.

Variables	Altruistic motivation	Career choice intentions	
	Model 1	Model 2	Model 3
Constant	1.523***	1.526***	0.925***
Gender	0.004	0.028	0.026
Age	−0.013	−0.040	−0.035
Household registration	−0.054	0.017	0.039
Families’ socioeconomic status	0.014	−0.037	−0.043
PSM	0.709***	0.635***	0.355***
Altruistic motivation			0.395***
Total effect [95% CI]		0.635 [0.530, 0.740]	
Direct effect [95% CI]		0.355 [0.237, 0.474]	
Indirect effect [95% CI]		0.280 [0.188, 0.374]	
*R* ^2^	0. 296***	0.190***	0.275***

*N* = 624, Bootstrap size = 5,000. CI = confidence interval. *p < 0.05, **p < 0.01, and ***p < 0.001.

To test the moderating role of professional values, this study employed the SPSS PROCESS macro, selecting Model 5 for verification. Table 5 shows that both PSM and professional values exert a significant positive impact on career choice intentions (b = 0.523, *p* < 0.001; b = 0.144, *p* < 0.001). Moreover, the interaction effect between PSM and professional values significantly positively affects career choice intentions (b = 0.096, *p* < 0.05). We divided professional values into low, medium, and high levels by adding and subtracting one standard deviation from the mean. The results indicate that under different levels of professional values, the direct effects are all statistically significant (b = 0.449, 95% CI: [0.322, 0.577]; b = 0.523, 95% CI: [0.403, 0.643]; b = 0.597, 95% CI: [0.452, 0.741], respectively). These results suggest that professional values have a positive moderating effect on the relationship between PSM and career choice intentions. Therefore, Hypothesis 3 is validated.

**Table 5:** Results of moderating hypotheses.

Variables	Career choice intentions
Constant	3.792***
Gender	−0.035
Age	−0.018
Household registration	−0.018
Families’ socioeconomic status	−0.039
PSM	0.523***
Professional values	0.144***
PSM x Professional values	0.096*
Conditional effects at values of the moderator [95% CI]	
M − SD (−0.769)	0.449 [0.322, 0.577]
Mean (0)	0.523 [0.403, 0.643]
M + SD (0.769)	0.597 [0.452, 0.741]
*R* ^2^	0.221

*N* = 624, Bootstrap = 5,000, *p < 0.05, **p < 0.01, and ***p < 0.001.

**Figure 2:**
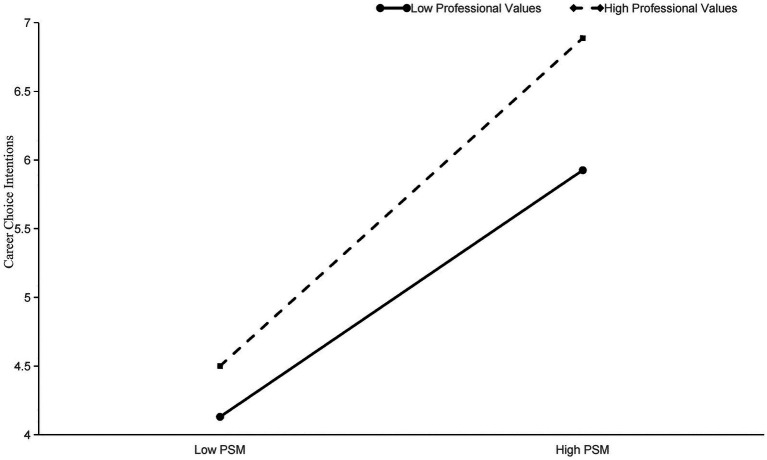
Interaction plot of PSM and professional values predicting career choice intentions.

To provide a clearer illustration of the moderating role of professional values, we drew the interaction plots (Figure 2). Figure 2 shows that the slope for the high group is notably steeper compared to that of the low group, suggesting that at higher levels of professional values, the impact of PSM on career choice intentions is stronger. In other words, the association between PSM and career choice intentions is positively moderated by professional values. Thus, Hypothesis 3 is further verified.

## 5 Discussions

This study explored the relationship between public service motivation (PSM) and career choice intentions among social work students, while also examining the mediating roles of altruistic motivation and professional values in how PSM influences career choices. First, the results confirm that PSM has a significant positive effect on social work students’ career choice intentions. Specifically, students with higher levels of PSM are more inclined to pursue careers in the public or nonprofit sectors. This finding is consistent with previous studies (Asseburg and Homberg, 2020; Hinna et al., 2021). According to Expectancy Theory, individuals with higher PSM are more likely to believe that public service careers align with their intrinsic needs and values, which in turn makes them more likely to choose such careers (Wanous et al., 1983). Although Public Service Motivation (PSM) has its roots in Western culture, it naturally aligns with Chinese traditional culture, particularly Confucianism, which emphasizes “benevolence” and “social responsibility.” In China, collectivism predominates, and the career choices of social work students may not be solely based on personal interests but also on a desire to contribute to the collective and society. Therefore, as an intrinsic motivation, PSM may have a stronger positive influence on social work students in China, encouraging them to pursue careers in the public service or nonprofit sectors.

Second, this study finds that altruistic motivation partially mediates the relationship between PSM and career choice intentions. The explanation is that altruistic motivation drives social work students toward careers that focus on public service, with PSM acting as a catalyst to strengthen these altruistic tendencies. The social structure, traditional culture, and value system of China profoundly influence individuals’ career decisions, particularly in the public service sector. In China, the cultural values of collectivism and social responsibility are deeply ingrained. In contrast to the individualism emphasized in Western cultures, Chinese people are more inclined to focus on the interests of family, society, and the nation. An individual’s public service motivation may initially be activated by their intrinsic altruistic motivation, which then influences their career intentions toward public service professions. For example, societal respect and admiration for altruistic professions can strengthen an individual’s public service motivation, further enhancing their desire to serve others and society, thus prompting them to choose these fields. Therefore, the mediating role of altruistic motivation in this process not only reflects an individual’s recognition of social responsibility but also embodies the high regard in Chinese culture for “working for the benefit of others.”

Third, the study indicates that professional values positively moderate the relationship between PSM and career choice intentions. In other words, students who strongly identify with altruistic professional values are more likely to experience a stronger positive impact of PSM on their career intentions. Because their professional values align closely with the intrinsic motivations of PSM, they view the public sector as a better avenue for realizing their personal values, thus increasing their likelihood of choosing public service-related professions (Holt, 2018). In China, a profession is not only a means of livelihood but also an important aspect of personal social identity and the embodiment of individual values. The traditional Chinese concept of “serving the country and the people,” along with the modern emphasis on “serving and contributing to society,” closely links public service motivation with the professional values of social work. When social work students choose to engage in public service, it is often driven not only by a sense of social responsibility but also by their identification with their own professional and moral values. In the process of career choice, social work students may place greater emphasis on realizing personal values and contributing to society and others.

### 5.1 Theoretical implications

This study makes several important theoretical contributions to the existing literature by examining the intricate mechanisms through which public service motivation (PSM) influences the career choice intentions of social work students. First, the findings highlight that PSM significantly increases the likelihood of social work students choosing careers in the public or nonprofit sectors. While research on PSM and career choice intentions remains debated (Lee and Choi, 2016), it is clear that PSM is recognized as a crucial antecedent in explaining career choices across different cultural contexts. This study confirms the positive effect of PSM on career choice intentions in the Chinese context, thus providing empirical evidence supporting the relationship between PSM and career choices and contributing to the growing body of literature on this topic. Since the social work profession originated in Western culture, it has not yet established a widely recognized theoretical foundation that integrates traditional Chinese culture. Therefore, this study aims to explore how public service motivation influences the career intentions of social work students, thereby deepening the understanding of professional identity in social work within the Chinese cultural context and promoting the localization of public service motivation theory in China.

Second, this research sheds light on the mediating mechanisms underlying the link between PSM and career choice intentions. Previous studies have primarily focused on the direct effects of PSM on career choices, with limited attention to the mediating factors. This study extends existing research by being the first to validate the partial mediating role of altruistic motivation in the relationship between PSM and career choice intentions, providing a deeper understanding of the underlying processes. Although Confucian culture in China also advocates “benevolence” as the highest moral standard, emphasizing personal care for others and collective values, particularly at the family, societal, and national levels, these altruistic cultures and altruism have been gradually weakened in the process of market-oriented development. Therefore, this study also contributes to further prompting academic attention and research on the theoretical role of traditional Chinese altruistic culture in the career choices of today’s young students.

Third, the study also uncovers the moderating mechanisms in the relationship between PSM and career choice intentions. While much of the previous research has emphasized the direct influence of professional values on career intentions (Ni et al., 2022), few studies have explored how professional values affect the relationship between PSM and career choices. Only a handful of scholars have investigated the moderating roles of variables like time and relational work (Wright and Christensen, 2010; Choi, 2017). Therefore, this study is the first to confirm the moderating role of professional values in the PSM-career intention link, thus advancing and enriching the literature on PSM theory. With the rapid development of Chinese society and the process of marketization, the career values of social work students may be influenced by factors such as modernization, globalization, and economic interests. This study reveals how career values influence social work students’ career intentions as they navigate the balance between serving others and the realities of societal development. It expands our theoretical understanding of how contemporary Chinese social work students balance ideals and reality in their career choices.

### 5.2 Practical implications

The present study provides valuable practical insights for supporting and guiding the career development of social work students. First, given that public service motivation (PSM) significantly enhances social work students’ likelihood of choosing careers in the public or non-profit sectors, educational institutions should take effective and specific measures to enhance social work students’ PSM. Specifically, social work educators can design curriculum content and case studies that foster a sense of social responsibility, helping students better understand the value and societal impact of public service, thereby strengthening their attention to the public good and sense of responsibility. In addition, educators should increase opportunities for students to engage in community service, government projects, or internships with non-profit organizations, further enhancing their sense of social responsibility and professional identity in social work. This, in turn, will stimulate their career intention to enter the public or non-profit sectors. Moreover, it is crucial for policymakers in China to recognize the developmental value of the social work profession and to provide more positions and career development opportunities in the public and non-profit sectors.

Second, the study highlights that PSM can indirectly affect career choice intentions by fostering altruistic motivation. Since altruistic motivation has a positive effect on career choices, universities should focus on nurturing this spirit within their social work programs. Although the social work profession originated in the West and is rooted in Western culture, Chinese traditional culture also contains prominent ideas of altruistic motivation. Therefore, social work educators can integrate elements of traditional Chinese culture into their classroom teaching to explore the cultural roots of social work education and development in China. For example, educators can promote the Confucian concept of “ren” (benevolence) in their teaching, guiding students to draw upon cultural traditions for the values of altruistic behavior. In Chinese culture, social morality and family obligations are regarded as important guides for individual behavior. Social work education can emphasize traditional Chinese social moral norms, such as “yi” (righteousness) and “li” (propriety), highlighting that serving others and helping vulnerable groups is a reflection of social responsibility. On the other hand, educators can offer social work students more opportunities to engage in community service, group activities, and volunteer work, allowing them to directly experience the impact of altruistic behavior on others, enhancing their sense of self-efficacy, and thereby strengthening their altruistic motivation.

Third, because professional values can strengthen the positive relationship between PSM and career choice intentions, universities should incorporate education on professional values into their curricula. Teaching students about the professional values that underpin social work, such as public service and social contribution, can help them establish a deeper connection to the profession. Career development planning in universities should be integrated into academic programs to help students understand the career paths and opportunities in social work, increasing their confidence and alignment with the profession’s core values. More importantly, in the context of marketization, it is essential for both the government and universities to guide students in establishing correct professional values through various channels, with a focus on the education of professional ethics and social responsibility. For instance, the government should promote the importance of noble professional ethics and advocate for a sense of social responsibility, especially in the public service sector, through policy guidance and public awareness campaigns. Universities can incorporate modules related to professional ethics and social responsibility into their curricula, helping students understand how to balance personal interests with social responsibility in a market-driven environment, thereby fostering positive professional values.

### 5.3 Limitations and future research

Although this study presents several innovations and improvements over previous research, it also has certain limitations. First, as a quantitative study based on self-reported survey data, the research relies entirely on processed data and lacks the depth provided by case interviews. For example, there are significant ethnic and cultural differences across China’s provinces and regions, which also lead to variations in career choice perceptions. Our study only surveyed social work students from three comprehensive universities in China, and thus, the findings may not fully represent the career choice intentions of social work students across all Chinese universities. This limitation restricts the generalizability of the findings and limits the development of more nuanced theoretical constructs. Future research should incorporate in-depth interviews or field studies with specific groups to enhance the scientific rigor and depth of the results. Second, this study used a Chinese-translated version of Perry’s Public Service Motivation (PSM) Scale, which was originally designed within a Western context. Scholars have noted that the scale’s structure may not be fully appropriate when applied to non-Western contexts, potentially affecting the validity of the results (Kim and Vandenabeele, 2010). Therefore, the findings of this study should be interpreted with caution. Western culture, particularly with its emphasis on individualism and public service ideals, prioritizes personal choice, freedom, and self-actualization. In contrast, Chinese culture is more collectivist, emphasizing family responsibility, social obligations, and the collective good. As a result, Perry’s (1996) Public Service Motivation (PSM) scale may not fully reflect the cultural values of Chinese students. In China, career decisions are often influenced not only by individual interests and achievements but also by societal expectations and family pressures—factors that may not be adequately captured by Western-based scales. Moreover, China’s multi-ethnic landscape introduces further complexity. Different ethnic groups bring unique cultural perspectives, which can limit the generalizability of our findings across the country. These cultural differences suggest that our results may not be easily applicable to all regions or ethnic communities within China. To address these issues, we will further adapt the Western PSM scale to better reflect Chinese cultural values in the future research. This could involve adding items related to collective responsibility and family expectations, providing a more comprehensive measure of public service motivation among Chinese students. Third, this study employed only cross-sectional data, which limits the ability to draw conclusions about causal relationships between variables. Without a longitudinal design, it is difficult to track changes over time and understand how the relationships between variables evolve. To address this limitation, future studies should consider using longitudinal or experimental data, which would provide more robust insights into the causal relationships between variables and the long-term effects of PSM on career choice intentions. For example, we could design a virtual scenario or case in an experimental classroom, allowing students to make decisions in the context of a specific public service situation. This experimental approach would help us more accurately understand the impact of PSM on social work students’ career choice intentions.

## 6 Conclusion

The primary objective of this study was to explore the impact of PSM, altruistic motivation, and professional values on the career choice intentions of social work students, with a focus on explaining the relationship between PSM and the career choice intentions of social work students. While previous studies have examined the influence of PSM on the work attitudes and behaviors of public sector employees (Holt, 2018; Asseburg and Homberg, 2020; Gan et al., 2020; Hinna et al., 2021), few studies have delved into the relationship between PSM and the career choice intentions of social work students. This study further confirmed the significant positive effect of PSM on the career choice intentions of social work students, as well as the mediating role of altruistic motivation and the moderating role of professional values in the relationship between these two factors. Consequently, this study deepens our understanding of the career choice motivations of social work students, demonstrating that PSM provides the impetus for career choices, altruistic motivation serves as the specific behavioral guidance for this drive, and professional values play a moderating role in the process. However, it is important to recognize the complexity of the causal relationship between PSM and career choice intentions, which may also be influenced by varying cultural and institutional contexts. Therefore, future research should further examine the application and validation of PSM theory from a cross-cultural perspective, particularly emphasizing the differential impact of traditional Confucian culture in Asia versus Western culture on PSM.

## Data Availability

The original contributions presented in the study are included in the article/Supplementary material, further inquiries can be directed to the corresponding author.

## Supplementary material

The Supplementary material for this article can be fund online at: https://www.frontiersin.org/articles/10.3389/fpsyg.2025.1517457/full#supplementary-material
